# Successful Management of Refractory Torsades De Pointes Due to Drug-Induced Long QT Syndrome Guided by Point-of-Care Monitoring of Ionized Magnesium

**DOI:** 10.7759/cureus.13939

**Published:** 2021-03-17

**Authors:** Chiho Matsuura, Takao Kato, Kaoru Koyama

**Affiliations:** 1 Department of Anesthesiology, Saitama Medical Center, Saitama Medical University, Kawagoe, JPN

**Keywords:** drug-induced long qt syndrome, torsades de pointes, ionized magnesium, magnesium sulfate

## Abstract

Ionized magnesium (iMg) is the physiologically active fraction, although total magnesium (tMg) is often used clinically because a dedicated electrode is required to measure the iMg concentration. The tMg concentration is not correlated with the iMg concentration, especially in severely ill patients. In this report, a case of refractory torsades de pointes (TdP) due to drug-induced long QT syndrome was successfully treated with high-dose magnesium sulfate guided by point-of-care monitoring of the iMg concentration. A woman in her 60s had taken osimertinib for two months to treat lung cancer. TdP occurred after the operation of a thoracic compression fracture under general anesthesia. She was diagnosed with drug-induced long QT syndrome. TdP continued, despite treatment with 6 g magnesium sulfate. The iMg value on the admission to the intensive care unit was 0.92 mmol/L, but TdP occurred intermittently and circulatory dynamics were unstable. After an additional intravenous administration of 1 g magnesium sulfate, continuous intravenous administration was initiated at 1 g/h. TdP terminated when the iMg concentration reached 1.31 mmol/L. Then, the target iMg was set to 1.3 mmol/L. The iMg concentration was measured every two hours to adjust the continuous dose of magnesium sulfate. Magnesium administration was tapered, and she was transferred to a general ward on the third day. She was discharged without complications on the 11th day. Point-of-care monitoring of the iMg concentration and observation of the patient’s clinical symptoms were important for the effective and safe treatment of TdP due to drug-induced long QT syndrome.

## Introduction

Magnesium is an important cation that is involved in more than 300 enzymatic activities in the human body. It exists in plasma in three separate fractions: ionized, protein-bound, and ligand-complexed. As with calcium, ionized magnesium (iMg) is the physiologically active fraction. But clinically, total Mg (tMg) is used as a substitute, because a dedicated electrode is required to measure iMg. The routine monitoring of ionized calcium by blood gas analyzers is widespread in many hospitals. On the contrary, the measurement of iMg has not been clinically standard, because almost all blood gas analyzers but one can’t measure iMg. Previous studies [1-3] indicate that 60%-70% of the total magnesium exists as an ionized fraction; the tMg concentration is not correlated with the iMg concentration in severely ill patients. The poor correlation between iMg and tMg concentrations is attributed to a change in blood pH, protein concentration, and negatively charged ligands. Thus, tMg is not a good predictive marker for iMg, and iMg needs to be measured directly. The use of tMg as a therapeutic index may result in unnecessary tests and excessive magnesium administration. It may also cause clinicians to miss hidden hypermagnesemia or hypomagnesemia [2].

Magnesium sulfate is recommended as a first-line drug for treating torsades de pointes (TdP). However, magnesium overdose may cause adverse clinical symptoms [4,5]. Thus, close monitoring of the serum magnesium level is necessary for critically ill patients. We experienced a case of TdP due to drug-induced long QT syndrome that was successfully treated with a high dose of magnesium sulfate guided by point-of-care monitoring of the iMg concentration.

## Case presentation

A woman in her 60s had been taking osimertinib (80 mg) for two months for the treatment of lung cancer. She had pre-existing QT prolongation on admission to our hospital. The corrected QT interval (i.e., QT interval/RR interval) was 0.486 second, but she had no other medical history, including syncope. Electrocardiography was not performed after osimertinib administration.

She underwent surgery under general anesthesia for a thoracic compression fracture at another hospital. She had been taking osimertinib internally until the day before surgery. Other drugs that cause QT prolongation were not used. No symptoms of arrhythmia or cardiac complications occurred before surgery.

**Figure 1:**
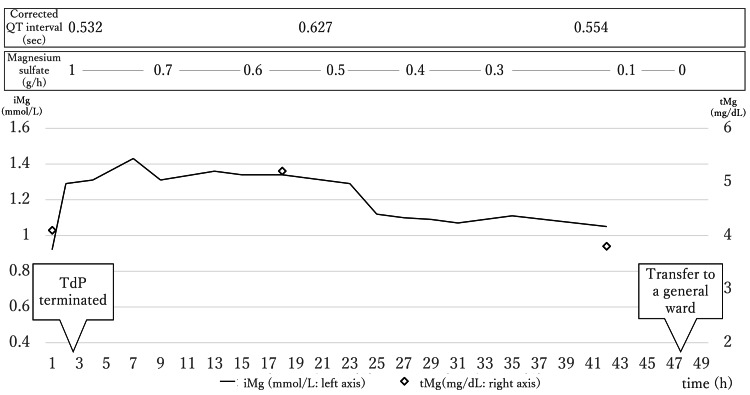
The patient’s clinical course after the ICU admission The patient’s clinical course after the ICU admission. Treatment was initiated with a magnesium sulfate bolus (1 g), followed by continuous infusion (at 1 g/h). TdP terminated approximately 90 minutes after starting the infusion of magnesium sulfate with an iMg concentration of 1.31 mmol/L. The solid line indicates the iMg level, and the open diamonds indicate the total magnesium level ICU intensive care unit, iMag ionized magnesium, tMg total magnesium, TdP torsades de pointes

On the night of the surgery, she experienced TdP with loss of consciousness. She was diagnosed with osimertinib-induced long QT syndrome. TdP continued, despite the administration of 6 g magnesium sulfate. Resuscitation including cardioversion was not performed because TdP was not persistent. She was transported to our hospital, and then admitted to the intensive care unit (ICU). Figure 1 shows the patient’s clinical course after the ICU admission.

At the time of admission to the ICU, the tMg concentration was 4.1 mg/dL, the iMg concentration was 0.92 mmol/L, and the potassium concentration was 4.4 mEq/L. The Stat Profile pHOx Ultra blood gas analyzer (Nova Biomedical, Waltham, MA, USA) was used to measure the iMg concentration.

**Figure 2:**
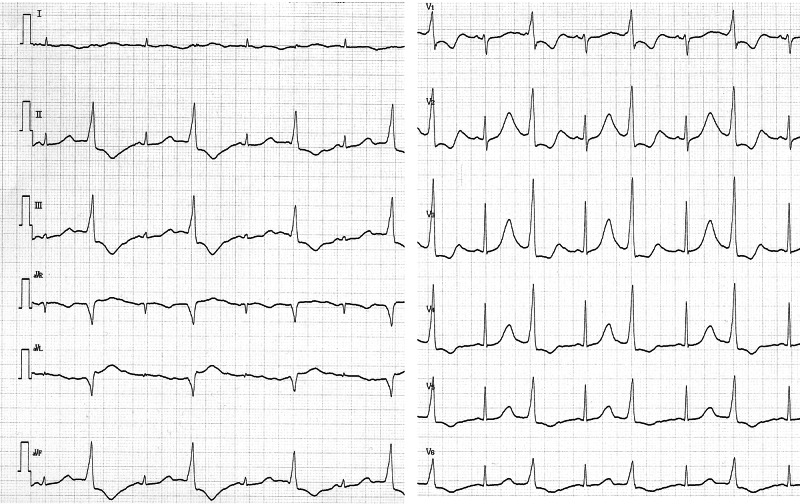
Twelve-lead electrocardiogram

**Figure 3:**
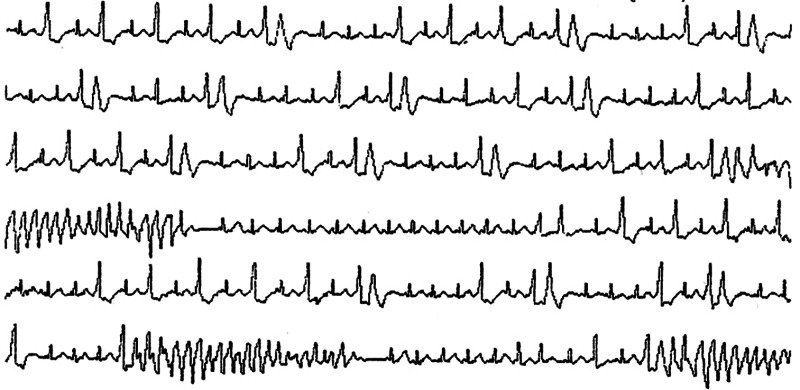
Lead II recording

The corrected QT interval was 0.532 second at the time. Despite the high iMg concentration (reference interval, 0.48-0.67 mmol/L), TdP occurred intermittently and her circulatory dynamics were unstable (Figures 2, 3).

We considered other treatments such as ventricular pacing and use of isoproterenol for the refractory TdP. However, many reports suggest that twice the upper limit of the tMg up to 5.0 mg/dL, which is estimated as 1.34 mmol/L of iMg, may be safe [6-8]. Therefore, after an additional intravenous administration of 1 g of magnesium sulfate, continuous intravenous administration was started at 1 g/h while the iMg concentration was monitored. The general protocol is to administer 2 g of magnesium and initiate its continuous administration at 10-20 mg/kg. We reduced the initial dose because the iMg concentration was high. Approximately 90 minutes after starting the continuous administration, TdP terminated. The iMg concentration at that time was 1.31 mmol/L. Thus, the target level of iMg was set to 1.3 mmol/L, and the iMg concentration was measured every two hours to adjust the continuous dose of magnesium sulfate.

On the second day of her ICU admission, magnesium administration was carefully tapered by 0.1-0.2 g/h while monitoring iMg concentrations, starting after the half-life of osimertinib when the effect of osimertinib was believed to have diminished. It was then discontinued on the third day. No improvement occurred in the corrected QT interval during the ICU stay. The final corrected QT interval was 0.554 seconds.

She was transferred to the general ward on the third day and discharged on the 11th day. The corrected QT interval was not measured at the time of discharge, but was measured in the outpatient clinic on day 12 after discharge and was 0.475 seconds. She had no clinical symptoms associated with the administration of high-dose magnesium during the course of treatment.

## Discussion

Osimertinib causes QT prolongation with a probability of 6.1% [9]. It needs to be administered more carefully in patients who already have QT prolongation. Our patient had slight QT prolongation (corrected QT interval = 0.486 seconds) before osimertinib administration; therefore, the QT interval was further prolonged in this patient after osimertinib administration. We expected the arrhythmia would continue after osimertinib was discontinued because of its long half-life (i.e., 48 hours) [10]. Therefore, the tapering of magnesium was initiated when the drug’s half-life period had elapsed after the discontinuation of the medication.

In addition, TdP occurred after surgery under general anesthesia. Investigators have reported that 80% of patients have QT prolongation immediately after general anesthesia [11]. Various causes of perioperative QT prolongation exist such as the use of drugs for which this is an adverse effect and surgical stress [11]. Investigators have also reported that QT prolongation occurs after general anesthesia, but not with local anesthesia [12]. In our patient, we speculate that, in addition to osimertinib administration, general anesthesia and perioperative stress caused further QT prolongation. For patients with QT prolongation, surgery under local or spinal anesthesia should be considered, if possible.

Treatment of TdP associated with drug-induced long QT syndrome involves first discontinuing the causative drug. Magnesium sulfate administration is recommended as the first treatment choice in addition to correcting the serum potassium level [13].

In our patient, the iMg/tMg ratio was 0.64, which was nearly the same value mentioned in previous reports [10]. In addition, tMg is a biochemical test parameter and its measurement requires 30-60 minutes in many hospitals. By contrast, iMg can be rapidly measured at the bedside by using a blood gas analyzer. The lengthy turnaround time of tMg is unsuitable whereas iMg is superior for critically ill patients under time-sensitive situations such as in the ICU. iMg is an especially useful parameter for proper dose adjustment and for the early detection of abnormal values. The relationship between iMg concentration and clinical symptoms are presented in Table 1.

**Table 1:** Magnesium levels and the corresponding clinical symptoms Assuming the iMg/tMg ratio = 0.64 [5] ECG electrocardiogram, iMg ionized magnesium, tMg total magnesium

tMg (mg/dL)	iMg (mmol/L)	Symptom
<1.2	<0.32	Neuromuscular irritability, arrhythmia, seizure, tetany
1.2–1.8	0.32–0.48	Hypokalemia, hypocalcemia
1.8–2.5	0.48–0.67	Adult normal range
2.5–5.0	0.67–1.34	Typically asymptomatic
5.0–7.0	1.34–1.86	Diminished deep tendon reflexes, nausea and vomiting, flushing, drowsiness, lethargy
7.0–12	1.86–3.2	ECG changes, hypotension, loss of deep tendon reflexes, somnolence
>12	>3.2	Coma, paralysis, cardiac arrest, complete heart block

Magnesium sulfate was administered to our patient at a previous hospital. However, TdP continued and her circulatory dynamics were unstable, even under high concentrations of tMg (4.1 mg/dL) and iMg (0.92 mmol/L). In general, patients may be asymptomatic up to 4.8 mg/dL of tMg, which is approximately two times higher than the upper limit of the normal range of tMg. Therefore, magnesium sulfate was additionally administered under the close monitoring of iMg. TdP was successfully treated without symptoms of hypermagnesemia by setting the target iMg concentration to 1.3 mmol/L. We only observed subjective symptoms in this patient, although deep tendon reflex assessments and subjective symptoms would have increased the safety of magnesium administration.

## Conclusions

We experienced a case of TdP, which was associated with perioperative drug-induced long QT syndrome, after surgery under general anesthesia. Guided by the monitoring of the iMg level, TdP was successfully treated without symptoms of hypermagnesemia. Thus, bedside monitoring of iMg and close observation of clinical symptoms can improve patient outcomes.
